# The Impact of Meteorology and Emissions on Surface Ozone in Shandong Province, China, during Summer 2014–2019

**DOI:** 10.3390/ijerph19116758

**Published:** 2022-06-01

**Authors:** Houwen Wang, Yang Gao, Lifang Sheng, Yuhang Wang, Xinran Zeng, Wenbin Kou, Mingchen Ma, Wenxuan Cheng

**Affiliations:** 1 College of Oceanic and Atmospheric Sciences, Ocean University of China, Qingdao 266100, China; wanghouwen@stu.ouc.edu.cn (H.W.); cengxinran@stu.ouc.edu.cn (X.Z.); 2 Frontiers Science Center for Deep Ocean Multispheres and Earth System, and Key Laboratory of Marine Environment and Ecology, Ministry of Education, Ocean University of China, and Qingdao National Laboratory for Marine Science and Technology, Qingdao 266100, China; kwb@stu.ouc.edu.cn (W.K.); mamingchen@stu.ouc.edu.cn (M.M.); chengwenxuan@stu.ouc.edu.cn (W.C.); 3 School of Earth and Atmospheric Sciences, Georgia Institute of Technology, Atlanta, GA 30332, USA; yuhang.wang@eas.gatech.edu

**Keywords:** ozone pollution, Shandong province, meteorology, ridges, biogenic emissions, ship emissions

## Abstract

China has been experiencing severe ozone pollution problems in recent years. While a number of studies have focused on the ozone-pollution-prone regions such as the North China Plain, Yangtze River Delta, and Pearl River Delta regions, few studies have investigated the mechanisms modulating the interannual variability of ozone concentrations in Shandong Province, where a large population is located and is often subject to ozone pollution. By utilizing both the reanalysis dataset and regional numerical model (WRF-CMAQ), we delve into the potential governing mechanisms of ozone pollution in Shandong Province—especially over the major port city of Qingdao—during summer 2014–2019. During this period, ozone pollution in Qingdao exceeded the tier II standard of the Chinese National Ambient Air Quality (GB 3095-2012) for 75 days. From the perspective of meteorology, the high-pressure ridge over Baikal Lake and to its northeast, which leads to a relatively low humidity and sufficient sunlight, is the most critical weather system inducing high-ozone events in Qingdao. In terms of emissions, biogenic emissions contribute to ozone enhancement close to 10 ppb in the west and north of Shandong Province. Numerical experiments show that the local impact of biogenic emissions on ozone production in Shandong Province is relatively small, whereas biogenic emissions on the southern flank of Shandong Province enhance ozone production and further transport northeastward, resulting in an increase in ozone concentrations over Shandong Province. For the port city of Qingdao, ship emissions increase ozone concentrations when sea breezes (easterlies) prevail over Qingdao, with the 95th percentile reaching 8.7 ppb. The findings in this study have important implications for future ozone pollution in Shandong Province, as well as the northern and coastal areas in China.

## 1. Introduction

China has been experiencing severe environmental pollution—particularly ozone pollution—in the summer. Although the Clean Air Action Plan of 2013 has led to reductions in the majority of air pollutant emissions [1], and subsequent decreases in PM_2.5_ concentrations [2,3,4], the trend of ozone concentrations is increasing [5,6]. Surface ozone may pose threats to human health [7,8] and plant growth [9,10]; thus, it is of vital importance to tackle the ozone pollution issue.

Surface ozone is produced mainly through complex photochemical reactions. Nitrogen oxides (NO_X_ ≡ NO + NO_2_) and volatile organic carbons (VOCs) are the primary precursors of ozone [11,12]. The O_3_–NOx–VOCs relationship can be understood through Empirical Kinetic Modeling Approach (EKMA) diagrams that delineate the different governance regimes of either VOC or NOx limitation [13,14]. Anthropogenic emissions—including industries, power plants, and mobile vehicles—are the major ozone-formation sources in megacities in China [14,15]. Meanwhile, biogenic VOCs are also vital for ozone production [16,17]. For instance, Wang et al. [18] conducted simulations with and without biogenic emissions, displaying that biogenic VOCs generally increase ozone concentrations by 5 ppb or less in and around large cities, but can reach 10–30 ppb in some areas. Biogenic VOCs can also affect ozone concentrations synergistically with anthropogenic emissions, reshaping the O_3_–NOx–VOC EKMA nonlinear relationship [19].

Ship emissions have non-negligible effects on air quality, especially over major ports [20,21,22]. Exhaust gases from ships (e.g., NOx and VOCs) affect the formation of ozone, particulate matter, as well as radical formation, in the atmosphere over the ocean and coastal cities [23,24,25]. For example, Wang et al. [26] suggest that ship emissions contribute to a maximum increase of 30–50 μgm^−3^ over the ship track areas in the Yangtze River Delta region of China. Emissions from ships passing through the port of Busan in Korea may directly affect O_3_ levels in coastal cities, and are estimated to increase ozone concentrations by up to 15 ppb at the coast and approximately 5 ppb inland due to photochemical production of gaseous pollutants [27]. In addition to emissions, ozone production is also sensitive to meteorological conditions, such as high temperature [28,29] and low wind speed [30]. Large-scale weather systems have continuous effects on ozone concentrations. For instance, the Western Pacific Subtropical High center over a specific region—with lower cloud cover, lower relative humidity, and stronger surface shortwave radiation—contributes to ozone formation in Shanghai by enhancing photochemical reactions [31]. However, it is unclear how a large-scale weather system causes high-ozone events in Shandong Province. Meanwhile, there are striking differences in climate between coastal and inland cities. Coastal cities are more susceptible to sea–land breezes [32], which can redistribute the pollutants.

This study focuses on the characteristics of ozone concentrations since the 2013 Clean Air Action Plan. Shandong is a coastal province in China with an international port city (Qingdao) and severe ozone pollution, selected to reveal the mechanisms of large-scale systems influencing the high-ozone events and the effects of emissions on ozone concentrations—especially ship emissions. Qingdao, situated in the southeast of Shandong Peninsula, and partly bordered by the Yellow Sea, is a major coastal port city in China with a population of approximately 10 million. We first describe model configurations and datasets in Section 2. The effects of meteorological conditions and emissions on ozone are then elucidated in Section 3, followed by conclusions and discussions in Section 4.

## 2. Model Descriptions and Datasets

Observations of maximum daily 8 h ozone concentration (MDA8) were downloaded from the China National Environmental Monitoring Centre (http://www.pm25.in, last access: 2 February 2022), with the majority of sites located in urban areas, and were interpolated to 0.5° × 0.5° grids to ease the analysis. The ERA5 reanalysis dataset [33] downloaded from the ECMWF (https://cds.climate.copernicus.eu/#!/home; accessed on 2 February 2022) was used to investigate the meteorological influences on the ozone concentrations, such as two-meter air temperature, surface shortwave radiation, and relative humidity.

**Figure 1:**
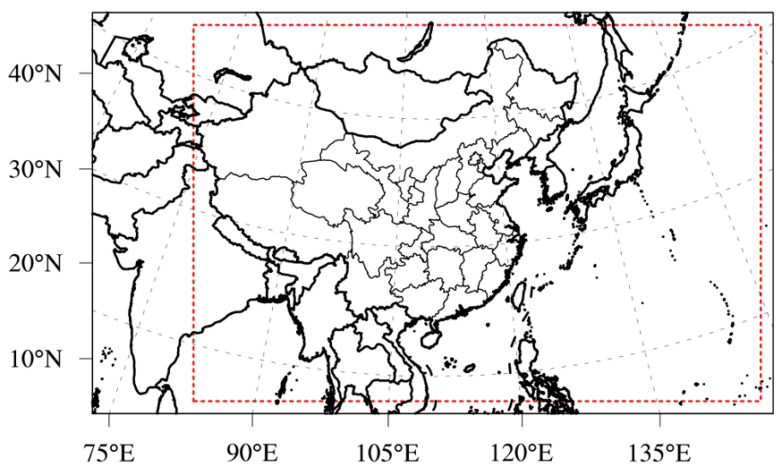
WRF-CMAQ domain. The black and red boxes represent the domains of WRF and CMAQ, respectively.

The meteorological conditions were simulated using the regional Weather Research and Forecasting model (WRF, version 4.1.1), with horizontal resolutions of 36 km × 36 km and 34 vertical layers. The model domain covered the entirety of China (Figure 1), with the center at 34° N, 110° E°. The boundary conditions and initial conditions of the WRF model were obtained from the 0.5° × 0.5° NCEP Climate Forecast System Version 2 [34]. Continuous simulations were performed annually from 2014 to 2019, with the previous December before each simulation discarded as spin-up. The physical parameterization schemes used in this study were based on our previous study [35,36], including the Rapid Radiative Transfer Model for GCMs (RRTMG) for shortwave and longwave radiation parameterization [37,38], the Grell–Freitas cumulus parameterizations [39], the Yonsei University (YSU) planetary boundary layer parameterizations [40], and the Morrison microphysics parameterization scheme [41].

This study uses the Community Multiscale Air Quality (CMAQ, version 5.3.1) model for atmospheric chemistry simulation. Aerosol Module Version 7 (AERO7) and Carbon Bond Version 6 (CB6) [42] represent the aerosol- and gas-phase chemistry. The Model for Ozone and Related Chemical Tracers Version 4 (MOZART-4) [43] was used to downscale chemical initial and boundary conditions (refer to Ma et al. [44] for more detailed information). The anthropogenic emission inventory of 2016 was taken from the 0.25° × 0.25° Multi-resolution Emission Inventory for China (MEIC, http://www.meicmodel.org; accessed on 2 February 2022) [45]. Ship emissions were calculated from the shipping emission inventory model [46,47]. The Model of Emissions of Gases and Aerosols from Nature (MEGAN, version 2.1) [48] was used to estimate hourly biogenic emissions. More information can be found in the work of Zeng et al. [35]. We also performed three sensitivity experiments with different emissions (Table 1) to explore the influence of emissions on ozone concentrations in Shandong Province. The biogenic contribution is defined as the simulation of SEN_ASB minus SEN_AS; the ship contribution is defined as the simulation of SEN_AS minus SEN_A; the contribution of biomass burning is defined as the simulation of CTRL minus SEN_ASB.

## 3. Results

### 3.1. Observed Ozone Characteristics

**Figure 2:**
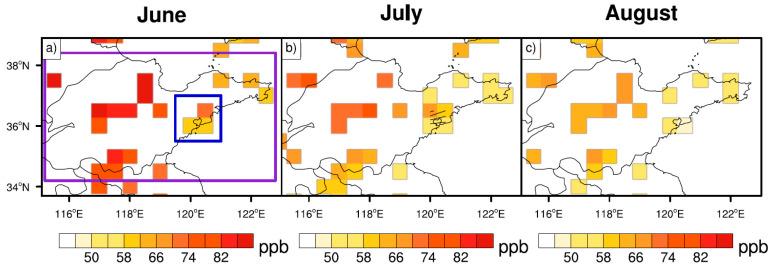
Monthly observations of MDA8 ozone concentrations (unit: ppb) at each grid in Shandong for (**a**) June, (**b**) July, and (**c**) August from 2014 to 2019. The blue and purple boxes in panel (**a**) denote Qingdao and Shandong Province, respectively.

The ozone distribution in Shandong Province (purple box in Figure 2a) was first calculated based on observations over each month during summer 2014–2019, as shown in Figure 2. In general, the ozone concentrations over Shandong Province were the highest in June, followed by July and August. The MDA8 ozone concentration exceeded 80 ppb in inland areas, such as parts of northwest Shandong Province. The seasonal average ozone concentration during summer 2014–2019 over the coastal city of Qingdao (the approximate colored area inside the blue box in Figure 2a) was 61.7 ppb—about 10 ppb lower than those in inland areas, likely associated with the relatively clean air from the ocean. As a result, ozone pollution was more severe in inland cities than on the coast.

**Figure 3:**
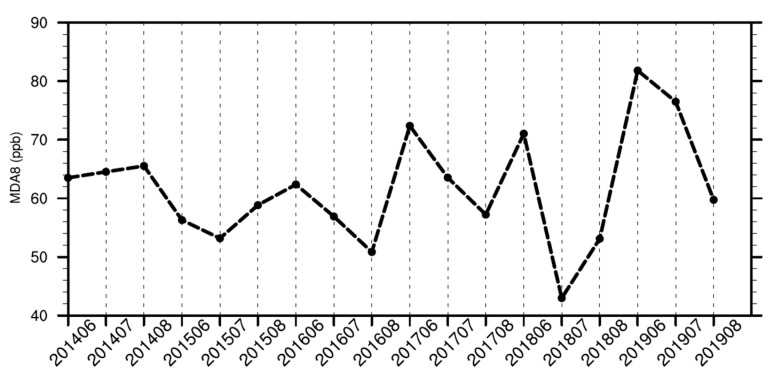
Monthly variations of observed MDA8 ozone concentrations (unit: ppb) in Qingdao during summer 2014–2019.

To elucidate the temporal characteristics of ozone pollution in Qingdao, the monthly variation of MDA8 ozone concentrations in Qingdao during summer 2014–2019 is presented in Figure 3. Compared with summer 2014–2018, ozone pollution in summer 2019 was extremely severe, reaching 81.8 ppb in June and 76.5 ppb in July. There were 30 days in Qingdao over the tier II standard of the Chinese National Ambient Air Quality GB 3095-2012 (160 μg/m^3^, i.e., 82 ppb at 298 K, 1 atm.) in the summer of 2019, compared with only a total of 45 days during the summers of 2014–2018. The more severe ozone pollution in Qingdao in June and July 2019 than in previous years is consistent with that in the North China Plain [49,50].

### 3.2. Meteorological Conditions Contributing to Ozone Pollution

In this section, the meteorological variables from the ERA5 dataset are averaged over Shandong Province and Qingdao. Table 2 displays the possible meteorological factors that may be connected with the daily variations of MDA8 ozone concentrations in Shandong Province during summer 2014–2019. The surface shortwave radiation and near-surface relative humidity at 1000 hPa are strongly correlated with MDA8 ozone concentrations in Shandong Province, with correlation coefficients of 0.67 and −0.66 during summer 2014–2019, respectively, stressing the crucial role of intense sunlight in photochemical reactions [51,52,53].

Moreover, regional transport is also an essential factor affecting ozone concentrations. Although the wind speed at 10 m shows no significant relationship with MDA8 ozone concentrations in Shandong Province, zonal wind speed at 10 m is closely associated with MDA8 ozone concentrations in Shandong Province, especially in Qingdao, with a correlation coefficient of 0.42 (displayed in Table 2). The correlation indicates that the stronger westerly can transport gaseous pollutants from the upstream, such as the North China Plain, to Qingdao, aggravating local ozone pollution, akin to regional ozone transport from the polluted Pearl River Delta to another coastal city—Hong Kong [54].

Notably, although the maximum daily temperature is significantly correlated with MDA8 ozone concentration in Qingdao, the correlation is lower than that based on surface shortwave radiation and relative humidity, likely due to the high relative humidity (i.e., 75% during summer 2014–2019) in coastal areas [52]. Therefore, the surface shortwave radiation and relative humidity are the most important factors affecting the ozone concentrations in Shandong Province. The correlation coefficient between maximum daily temperature and ozone concentrations is smaller in coastal cities than in inland cities.

To delve into the large-scale weather systems influencing ozone pollution in summer, a simple method was designed to identify high-ozone events objectively. A high-ozone event is detected when ozone concentrations in an area exceed 82 ppb (tier II standard of the Chinese National Ambient Air Quality GB 3095-2012) for at least four consecutive days. During the summer of 2014–2019, five high-ozone events were identified in Qingdao, occurring at 7 June–10 June 2017, 21 June–24 June 2018, 1 June–4 June 2019, 12 July–16 July 2019, and 19 August–22 August 2019. These all took place in the latter three years (2017–2019), with three during the summer of 2019, further highlighting the trend of increasing ozone in recent years.

**Figure 4:**
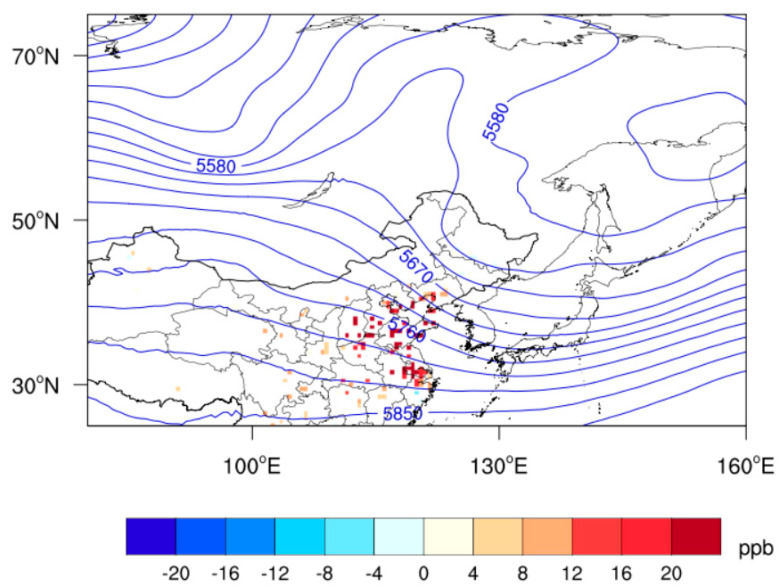
Composite geopotential heights (blue contour, units: m) at 500 hPa and observed MDA8 ozone anomalies (units: ppb) compared to the summer mean of 2014–2019 for high-ozone events in Qingdao during summer 2014–2019.

Geopotential height at 500 hPa and ozone anomalies compared with the summer mean of 2014–2019 were composited for five high-ozone events in Qingdao to delineate the atmospheric circulations (Figure 4). A conspicuous high-pressure ridge dominated over Lake Baikal and its northeast, concurrent with a cyclonic system over northern Japan and Northeast China. Controlled by the high-pressure ridge, the relative humidity and surface shortwave radiation anomalies in Qingdao during high-ozone events were −12% and 39 W/m^2^, respectively, compared with the summer mean during 2014–2019, conducive to the occurrence of high-ozone events.

### 3.3. Contribution of Different Emissions to Ozone Concentrations

The observed data were first used to evaluate the meteorological factors in WRF-CMAQ during summer 2014–2019, e.g., hourly air temperature at 2 m, wind direction at 10 m, and wind speed at 10 m (Table 3). The performance of the WRF model was reasonable over Shandong Province, generally meeting the benchmark [55], except for the mean gross of wind direction at 10 m. Meanwhile, the relatively large gross error in wind direction at 10 m—i.e., 44.0° for Shandong Province and 46.8° for Qingdao—may be caused by values close to 0 or 360 degrees [56].

**Figure 5:**
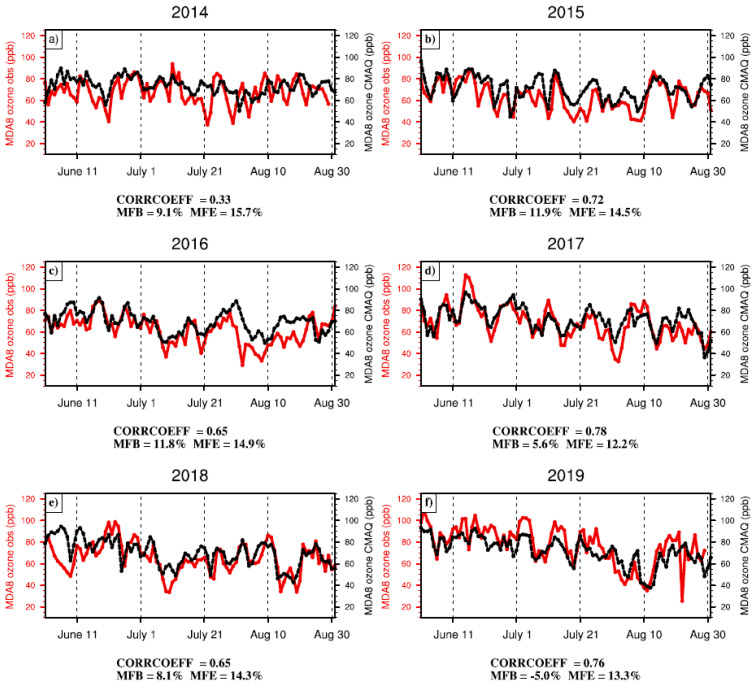
Comparison of MDA8 ozone concentrations over Shandong Province in each summer. Panels (**a**–**f**) show the MDA8 ozone concentrations from 2014 to 2019. The red and black lines denote observations and the CTRL simulation, respectively. CORRCOEFF, MFB, and MFE represent the correlation coefficient, mean fractional bias, and mean fractional error, respectively.

The model evaluation was also conducted between observed and simulated MDA8 ozone concentrations in Shandong Province each year during summer 2014–2019 (Figure 5). The mean fractional bias (MFB) and mean fractional error (MFE) during 2014–2019 were less than the benchmarks of 15% and 35% applied from a previous study [57], respectively, indicating that the simulated MDA8 ozone concentrations in the CTRL experiment can reproduce the observed MDA8 ozone concentrations in Shandong Province.

**Figure 6:**
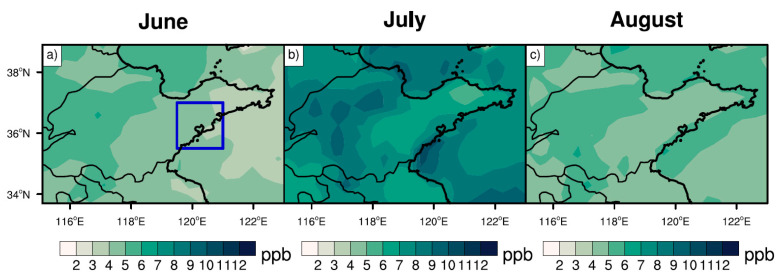
Monthly MDA8 ozone concentrations (units: ppb) over Shandong Province in (**a**) June, (**b**) July, and (**c**) August during 2014–2019, caused by biogenic emissions. The blue box in Figure 6a denotes Qingdao.

Figure 6 shows the MDA8 ozone concentrations from biogenic emissions (see Section 2) during summer 2014–2019. The ozone enhancement caused by biogenic emissions was highest in July—close to 10 ppb in the west and north of Shandong Province. The ozone concentrations in the east and south of Shandong—particularly coastal cities—were relatively low, ranging from 3 to 5 ppb. In June and August, the ozone concentrations from biogenic emissions were relatively uniform in coastal and inland areas, ranging from 3 to 4 ppb. The concentrations in the inland area were about 1 ppb higher than those in the coastal area. The seasonal average of ozone concentrations in Qingdao from biogenic emissions during summer 2014–2019 was 5.5 ppb, with the higher percentile—i.e., 95th percentile—reaching 15.5 ppb, further aggravating the ozone pollution in Qingdao. Please note that the selection of the 95th percentile is traditionally common in previous studies [44,58].

**Figure 7:**
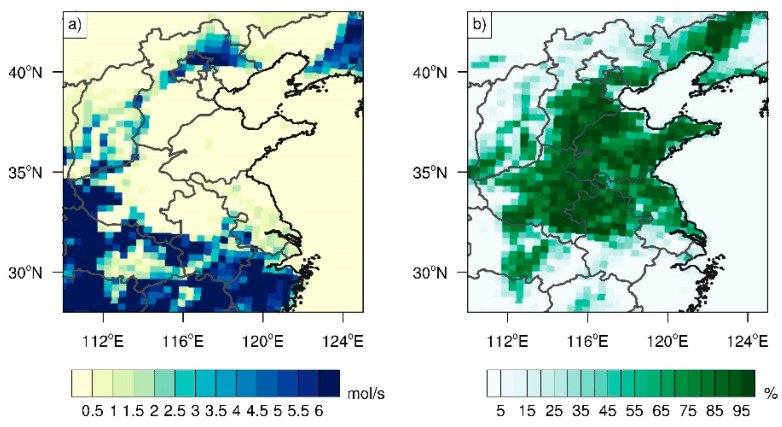
(**a**) Summer mean isoprene emissions (units: mol/s) during 2014–2019. (**b**) Spatial distribution of crops based on the land cover in 2016 from Moderate-Resolution Imaging Spectroradiometer (MODIS) data [62].

Considering that isoprene is generally the main biogenic VOC contributing to ozone production [59,60], we plotted the isoprene emissions during summer 2014–2019 (Figure 7a), finding that the isoprene emissions in the majority of Shandong Province were relatively low, i.e., 0.2 mol/s or less during summer 2014–2019 (Figure 7a). In contrast, regions around Shandong Province—such as the North China Plain and the Yangtze River Delta—had relatively high isoprene emissions. The primary reason for the low isoprene emissions in Shandong Province lies in the dominant vegetation type of crops (Figure 7b), which mainly emit methanol and other reactive VOCs, but have low isoprene emission rates [48,61].

**Figure 8:**
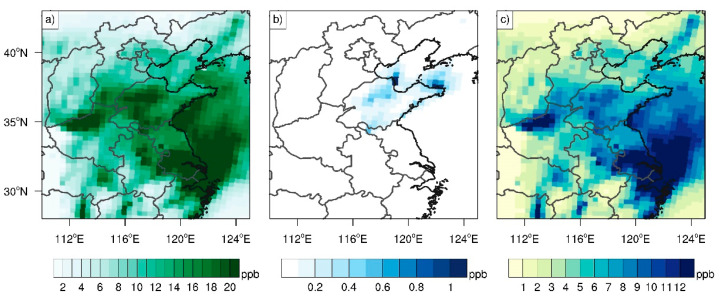
MDA8 ozone concentrations (units: ppb) caused by (**a**) biogenic emissions, (**b**) isoprene emissions in Shandong Province, and (**c**) isoprene emissions outside Shandong Province from 18 July 28 July 2017.

To unveil the effects of isoprene emissions—either in or outside Shandong Province—on ozone pollution in Shandong Province, the ozone episode (18–28 July 2017) with the largest contribution from isoprene emissions during 2014–2019—i.e., more than 10 ppb averaged in Shandong Province continuously for 11 days (Figure 8a)—was selected. We designed two additional scenarios during this period, by turning off the isoprene emissions in and outside Shandong Province. These two scenarios were each compared with the case SEN_ASB, so as to elucidate the effects of isoprene emissions in or outside of Shandong Province on ozone concentrations in Shandong Province.

The contribution of isoprene emissions in Shandong to MDA8 ozone concentrations therein was generally less than 1 ppb during this period (Figure 8b), due to low isoprene emissions, whereas natural forests in southern China emit large amounts of isoprene [63], accounting for approximately half of ozone enhancement from biogenic VOCs in southern China (Figure 8a vs. Figure 8c). Moreover, the enhanced ozone outside Shandong Province due to isoprene emissions is likely transported to Shandong Province (Figure 8c), with the detailed transport pathway discussed below.

**Figure 9:**
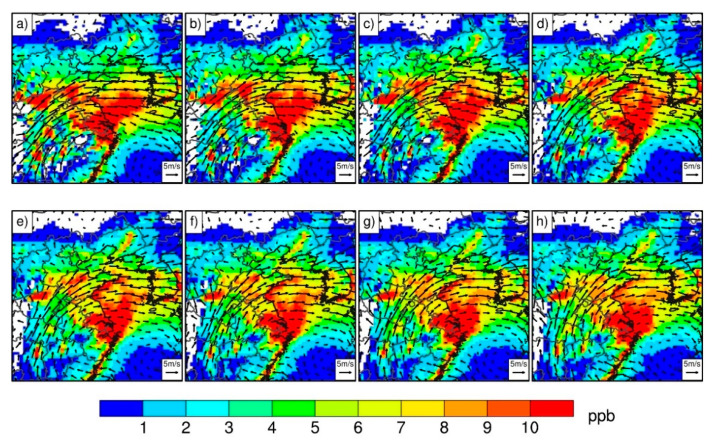
Composite hourly ozone concentrations (shading, units: ppb) from isoprene emissions outside Shandong Province and 850 hPa wind vectors (units: m/s) at (**a**–**h**) 09:00 to 16:00 local time from 18 July to 28 July 2017.

To reveal the effect of regional transport on the ozone concentrations contributed by biogenic emissions in Shandong Province, the hourly ozone concentrations during 09:00–16:00 local time due to isoprene emissions outside Shandong Province, as well as an 850 hPa wind vector, were composited during 18 July to 28 July 2017, and are presented in Figure 9. During these 11 days, eastern China was controlled by the Northwest Pacific Subtropical High. Dominated by the prevailing southwesterly wind, the ozone produced by biogenic emissions was transported from the southern flank of Shandong Province to Shandong Province. Therefore, biogenic emissions in regions around Shandong Province can affect Shandong Province through regional transport under favorable meteorological conditions.

**Figure 10:**
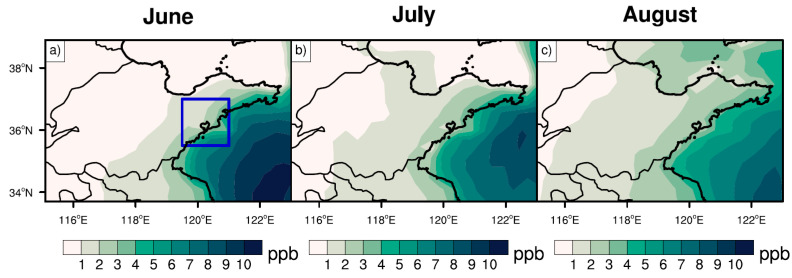
Monthly MDA8 ozone concentrations (units: ppb) over Shandong Province in (**a**) June, (**b**) July, and (**c**) August during 2014–2019, caused by ship emissions. The blue box in (**a**) denotes Qingdao.

The distribution of MDA8 ozone concentrations contributed by ship emissions (see Section 2) was analyzed in Shandong Province and the nearby seas (Figure 10). Ozone concentrations affected by ship emissions over the Yellow Sea were the highest in June, followed by July and August. In June, the MDA8 ozone concentrations over the Yellow Sea exceeded 10 ppb, gradually decreasing over the land, such as over Shandong Province. In terms of the land areas affected by ship emissions, the largest period occurred in August. Zooming into the port city of Qingdao, the average MDA8 ozone concentrations due to ship emissions were 2.4 ppb, with the 95th percentile of 8.7 ppb under favorable wind directions. In particular, when easterlies are dominant, ship emissions are prone to enhancing ozone concentrations in Qingdao.

Overall, biogenic and ship emissions play important roles in ozone concentration enhancement under favorable weather conditions, paving the way for ozone control and prediction. Under a changing climate—in particular of the increase in the frequency and intensity of extreme weather events, i.e., heat waves and stagnation [58,64]—it is of great importance to further examine how the interactions between extreme weather events and emissions—such as biogenic emissions—modulate the future ozone concentrations, as well as the associated uncertainties in constraining the signal in future changes.

## 4. Conclusions and Discussions

This study investigated the underlying mechanisms affecting the surface ozone concentrations in Shandong Province, including the major port city of Qingdao, during summer 2014–2019, by utilizing observation, ERA5 reanalysis datasets, and WRF-CMAQ modeling. The summer ozone concentrations in Qingdao were the highest in June, consistent with but lower than those in other inland cities in Shandong Province. The MDA8 ozone concentrations over Qingdao during summer 2014–2019 were highest in 2019, with 30 days exceeding 82 ppb, in contrast to an average of 9 days during summers in 2014–2018.

Surface shortwave radiation and relative humidity were most closely related to MDA8 ozone concentrations in Shandong Province. The high-pressure ridge appearing over Lake Baikal and to its northeast resulted in relatively low humidity and sufficient sunlight in Qingdao, critical for the occurrence of local high-ozone events.

The ozone concentrations contributed by biogenic emissions were highest in July during 2014–2019—close to 10 ppb in the west and north of Shandong Province. Numerical experiments indicate that the local impact of biogenic emissions on ozone in Shandong Province is relatively small. In contrast, the biogenic emissions over the southern flank of Shandong Province trigger ozone enhancement and further transport northeastward, leading to an increase in ozone concentration in Shandong Province. Additionally, in Qingdao, the ozone concentrations due to ship emissions are associated with sea breeze. The summer average ozone concentrations from ship emissions were 2.4 ppb in Qingdao during 2014–2019, with the 95th percentile of 8.7 ppb.

## Figures and Tables

**Table 1:** Emission settings of different sensitivity experiments using WRF-CMAQ.

Experiment	CTRL	SEN_A	SEN_AS	SEN_ASB
Anthropogenic emissions	Yes	Yes	Yes	Yes
Ship emissions	Yes	No	Yes	Yes
Biogenic emissions	Yes	No	No	Yes
Biomass burning emissions	Yes	No	No	No

**Table 2:** Correlation coefficients between MDA8 ozone concentrations and possible meteorological factors over land from the ERA5 dataset during summer 2014–2019.

	SSR	RH	U10	Tmax	WS10
Qingdao	0.50 *	−0.53 *	0.42 *	0.24 *	−0.13
Shandong	0.67 *	−0.66 *	0.25 *	0.44 *	−0.05

* indicates significance at *p* = 0.01. SSR, RH, U10, Tmax, and WS10 represent surface shortwave radiation, relative humidity, the u component of wind speed at 10 m, maximum daily temperature, and wind speed at 10 m.

**Table 3:** Evaluation of WRF simulation. OBS represents data from observations. Model represents results from controlled experiments in the WRF-CMAQ model.

Variables		Qingdao	Shandong Province	Benchmarks
Air temperature at 2 m (°C)	Mean OBS	25.35	25.84	
	Mean model	24.35	25.66	
	Mean bias	−1.00	−0.18	≤±0.5
	Gross error	1.80	1.63	≤2
Wind direction at 10 m (°)	Mean OBS	168.5	162.8	
	Mean model	172.3	161.1	
	Mean bias	3.7	−1.7	≤±10
	Gross error	46.7	44.0	≤30
Wind Speed at 10 m (m/s)	Mean OBS	3.27	3.15	
	Mean model	3.49	3.28	
	Mean bias	0.22	0.13	≤±0.5
	Gross error	1.20	1.18	

## Data Availability

The ERA5 reanalysis dataset was downloaded from the ECMWF (https://cds.climate.copernicus.eu/cdsapp#!/dataset/reanalysis-era5-single-levels-preliminary-back-extension?tab=overview; accessed on 2 February 2022). Ozone observations were obtained from the China National Environmental Monitoring Centre (http://www.pm25.in; accessed on 2 February 2022). Other data are available upon request from the corresponding author (yanggao@ouc.edu.cn).

## References

[1] Chinese State Council Action Plan on Air Pollution Prevention and Control. http://www.gov.cn/zwgk/2013-09/12/content_2486773.htm.

[2] Wang J., Zhao B., Wang S., Yang F., Xing J., Morawska L., Ding A., Kulmala M., Kerminen V.-M., Kujansuu J. (2017). Particulate matter pollution over China and the effects of control policies. Sci. Total Environ..

[3] Lin C.Q., Liu G., Lau A.K.H., Li Y., Li C.C., Fung J.C.H., Lao X.Q. (2018). High-resolution satellite remote sensing of provincial PM2.5 trends in China from 2001 to 2015. Atmos. Environ..

[4] Gao Y., Zhang L., Zhang G., Yan F., Zhang S., Sheng L., Li J., Wang M., Wu S., Fu J.S. (2020). The climate impact on atmospheric stagnation and capability of stagnation indices in elucidating the haze events over North China Plain and Northeast China. Chemosphere.

[5] Dang R., Liao H., Fu Y. (2021). Quantifying the anthropogenic and meteorological influences on summertime surface ozone in China over 2012–2017. Sci. Total Environ..

[6] Ma Z., Xu J., Quan W., Zhang Z., Lin W., Xu X. (2016). Significant increase of surface ozone at a rural site, north of eastern China. Atmos. Chem. Phys..

[7] Alexis N.E., Carlsten C. (2014). Interplay of air pollution and asthma immunopathogenesis: A focused review of diesel exhaust and ozone. Int. Immunopharmacol..

[8] Goodman J.E., Prueitt R.L., Sax S.N., Pizzurro D.M., Lynch H.N., Zu K., Venditti F.J. (2015). Ozone exposure and systemic biomarkers: Evaluation of evidence for adverse cardiovascular health impacts. Crit. Rev. Toxicol..

[9] Feng Z., Hu E., Wang X., Jiang L., Liu X. (2015). Ground-level O3 pollution and its impacts on food crops in China: A review. Environ. Pollut..

[10] McKee I.F., Long S.P. (2001). Plant growth regulators control ozone damage to wheat yield. New Phytol..

[11] Sillman S. (1995). The use of NO y, H2O2, and HNO3 as indicators for ozone-NOx-hydrocarbon sensitivity in urban locations. J. Geophys. Res.-Atmos..

[12] Atkinson R. (2000). Atmospheric chemistry of VOCs and NOx. Atmos. Environ..

[13] Sillman S. (1999). The relation between ozone, NOx and hydrocarbons in urban and polluted rural environments. Atmos. Environ..

[14] Ou J., Yuan Z., Zheng J., Huang Z., Shao M., Li Z., Huang X., Guo H., Louie P.K.K. (2016). Ambient Ozone Control in a Photochemically Active Region: Short-Term Despiking or Long-Term Attainment?. Environ. Sci. Technol..

[15] Li K., Jacob D.J., Liao H., Shen L., Zhang Q., Bates K.H. (2019). Anthropogenic drivers of 2013-2017 trends in summer surface ozone in China. Proc. Natl. Acad. Sci. USA.

[16] Li N., He Q., Greenberg J., Guenther A., Li J., Cao J., Wang J., Liao H., Wang Q., Zhang Q. (2018). Impacts of biogenic and anthropogenic emissions on summertime ozone formation in the Guanzhong Basin, China. Atmos. Chem. Phys..

[17] Geng F., Tie X., Guenther A., Li G., Cao J., Harley P. (2011). Effect of isoprene emissions from major forests on ozone formation in the city of Shanghai, China. Atmos. Chem. Phys..

[18] Wang Q.G., Han Z., Wang T., Zhang R. (2008). Impacts of biogenic emissions of VOC and NOx on tropospheric ozone during summertime in eastern China. Sci. Total Environ..

[19] Gao Y., Yan F., Ma M., Ding A., Liao H., Wang S., Wang X., Zhao B., Cai W., Su H. (2021). Unveiling the dipole synergic effect of biogenic and anthropogenic emissions on ozone concentrations. Sci. Total Environ..

[20] Endresen Ø., Sørgård E., Sundet J.K., Dalsøren S.B., Isaksen I.S.A., Berglen T.F., Gravir G. (2003). Emission from international sea transportation and environmental impact. J. Geophys. Res.-Atmos..

[21] Dore A.J., Vieno M., Tang Y.S., Dragosits U., Dosio A., Weston K.J., Sutton M.A. (2007). Modelling the atmospheric transport and deposition of sulphur and nitrogen over the United Kingdom and assessment of the influence of SO2 emissions from international shipping. Atmos. Environ..

[22] Zhang Y., Yang X., Brown R., Yang L., Morawska L., Ristovski Z., Fu Q., Huang C. (2017). Shipping emissions and their impacts on air quality in China. Sci. Total Environ..

[23] Eyring V., Isaksen I.S.A., Berntsen T., Collins W.J., Corbett J.J., Endresen O., Grainger R.G., Moldanova J., Schlager H., Stevenson D.S. (2010). Transport impacts on atmosphere and climate: Shipping. Atmos. Environ..

[24] Vutukuru S., Dabdub D. (2008). Modeling the effects of ship emissions on coastal air quality: A case study of southern California. Atmos. Environ..

[25] Feng J., Zhang Y., Li S., Mao J., Patton A.P., Zhou Y., Ma W., Liu C., Kan H., Huang C. (2019). The influence of spatiality on shipping emissions, air quality and potential human exposure in the Yangtze River Delta/Shanghai, China. Atmos. Chem. Phys..

[26] Wang R., Tie X., Li G., Zhao S., Long X., Johansson L., An Z. (2019). Effect of ship emissions on O3 in the Yangtze River Delta region of China: Analysis of WRF-Chem modeling. Sci. Total Environ..

[27] Song S.-K., Shon Z.-H., Kim Y.-K., Kang Y.-H., Oh I.-B., Jung C.-H. (2010). Influence of ship emissions on ozone concentrations around coastal areas during summer season. Atmos. Environ..

[28] Gao Y., Fu J.S., Drake J.B., Lamarque J.F., Liu Y. (2013). The impact of emission and climate change on ozone in the United States under representative concentration pathways (RCPs). Atmos. Chem Phys..

[29] Han H., Liu J., Shu L., Wang T., Yuan H. (2020). Local and synoptic meteorological influences on daily variability in summertime surface ozone in eastern China. Atmos. Chem. Phys..

[30] Jacob D.J., Winner D.A. (2009). Effect of climate change on air quality. Atmos. Environ..

[31] Chang L., Xu J., Tie X., Gao W. (2019). The impact of Climate Change on the Western Pacific Subtropical High and the related ozone pollution in Shanghai, China. Sci. Rep..

[32] Hai S., Miao Y., Sheng L., Wei L., Chen Q. (2018). Numerical Study on the Effect of Urbanization and Coastal Change on Sea Breeze over Qingdao, China. Atmosphere.

[33] Hersbach H., Bell B., Berrisford P., Hirahara S., Horányi A., Muñoz-Sabater J., Nicolas J., Peubey C., Radu R., Schepers D. (2020). The ERA5 global reanalysis. Q. J. R. Meteorol. Soc..

[34] Saha S., Moorthi S., Wu X., Wang J., Nadiga S., Tripp P., Behringer D., Hou Y.-T., Chuang H.-y., Iredell M. (2014). The NCEP Climate Forecast System Version 2. J. Clim..

[35] Zeng X., Gao Y., Wang Y., Ma M., Zhang J., Sheng L. (2022). Characterizing the distinct modulation of future emissions on summer ozone concentrations between urban and rural areas over China. Sci. Total Environ..

[36] Yan F., Gao Y., Ma M., Liu C., Ji X., Zhao F., Yao X., Gao H. (2021). Revealing the modulation of boundary conditions and governing processes on ozone formation over northern China in June 2017. Environ. Pollut..

[37] Iacono M.J., Delamere J.S., Mlawer E.J., Shephard M.W., Clough S.A., Collins W.D. (2008). Radiative forcing by long-lived greenhouse gases: Calculations with the AER radiative transfer models. J. Geophys. Res.-Atmos..

[38] Morcrette J.J., Barker H.W., Cole J.N.S., Iacono M.J., Pincus R. (2008). Impact of a New Radiation Package, McRad, in the ECMWF Integrated Forecasting System. Mon. Weather Rev..

[39] Grell G.A., Freitas S.R. (2014). A scale and aerosol aware stochastic convective parameterization for weather and air quality modeling. Atmos. Chem. Phys..

[40] Hong S.-Y., Noh Y., Dudhia J. (2006). A New Vertical Diffusion Package with an Explicit Treatment of Entrainment Processes. Mon. Weather Rev..

[41] Morrison H., Thompson G., Tatarskii V. (2009). Impact of Cloud Microphysics on the Development of Trailing Stratiform Precipitation in a Simulated Squall Line: Comparison of One- and Two-Moment Schemes. Mon. Weather Rev..

[42] Murphy B.N., Woody M.C., Jimenez J.L., Carlton A.M.G., Hayes P.L., Liu S., Ng N.L., Russell L.M., Setyan A., Xu L. (2017). Semivolatile POA and parameterized total combustion SOA in CMAQv5.2: Impacts on source strength and partitioning. Atmos. Chem. Phys..

[43] Emmons L.K., Walters S., Hess P.G., Lamarque J.F., Pfister G.G., Fillmore D., Granier C., Guenther A., Kinnison D., Laepple T. (2010). Description and evaluation of the Model for Ozone and Related chemical Tracers, version 4 (MOZART-4). Geosci. Model. Dev..

[44] Ma M., Gao Y., Wang Y., Zhang S., Leung L.R., Liu C., Wang S., Zhao B., Chang X., Su H. (2019). Substantial ozone enhancement over the North China Plain from increased biogenic emissions due to heat waves and land cover in summer 2017. Atmos. Chem. Phys..

[45] Li M., Zhang Q., Kurokawa J.I., Woo J.H., He K., Lu Z., Ohara T., Song Y., Streets D.G., Carmichael G.R. (2017). MIX: A mosaic Asian anthropogenic emission inventory under the international collaboration framework of the MICS-Asia and HTAP. Atmos. Chem. Phys..

[46] Liu H., Fu M., Jin X., Shang Y., Shindell D., Faluvegi G., Shindell C., He K. (2016). Health and climate impacts of ocean-going vessels in East Asia. Nat. Clim. Chang..

[47] Liu H., Meng Z.-H., Lv Z.-F., Wang X.-T., Deng F.-Y., Liu Y., Zhang Y.-N., Shi M.-S., Zhang Q., He K.-B. (2019). Emissions and health impacts from global shipping embodied in US–China bilateral trade. Nat. Sustain..

[48] Guenther A.B., Jiang X., Heald C.L., Sakulyanontvittaya T., Duhl T., Emmons L.K., Wang X. (2012). The Model of Emissions of Gases and Aerosols from Nature version 2.1 (MEGAN2.1): An extended and updated framework for modeling biogenic emissions. Geosci. Model. Dev..

[49] Li K., Jacob D.J., Shen L., Lu X., De Smedt I., Liao H. (2020). Increases in surface ozone pollution in China from 2013 to 2019: Anthropogenic and meteorological influences. Atmos. Chem. Phys..

[50] Ma X., Huang J., Zhao T., Liu C., Zhao K., Xing J., Xiao W. (2021). Rapid increase in summer surface ozone over the North China Plain during 2013–2019: A side effect of particulate matter reduction control?. Atmos. Chem. Phys..

[51] Pu X., Wang T.J., Huang X., Melas D., Zanis P., Papanastasiou D.K., Poupkou A. (2017). Enhanced surface ozone during the heat wave of 2013 in Yangtze River Delta region, China. Sci. Total Environ..

[52] Li M., Yu S., Chen X., Li Z., Zhang Y., Wang L., Liu W., Li P., Lichtfouse E., Rosenfeld D. (2021). Large scale control of surface ozone by relative humidity observed during warm seasons in China. Environ. Chem. Lett..

[53] Cheng F.-Y., Jian S.-P., Yang Z.-M., Yen M.-C., Tsuang B.-J. (2015). Influence of regional climate change on meteorological characteristics and their subsequent effect on ozone dispersion in Taiwan. Atmos. Environ..

[54] Chan C.Y., Chan L.Y. (2000). Effect of meteorology and air pollutant transport on ozone episodes at a subtropical coastal Asian city, Hong Kong. J. Geophys. Res.-Atmos..

[55] Emery C., Tai E. (2001). Enhanced Meteorological Modeling and Performance Evaluation for Two Texas Ozone Episodes.

[56] Zhang G., Gao Y., Cai W., Leung L.R., Wang S., Zhao B., Wang M., Shan H., Yao X., Gao H. (2019). Seesaw haze pollution in North China modulated by the sub-seasonal variability of atmospheric circulation. Atmos. Chem. Phys..

[57] EPA (2007). Guidance on the Use of Models and Other Analyses for Demonstrating Attainment of Air Quality Goals for Ozone, PM2.5, and Regional Haze.

[58] Zhang J., Gao Y., Luo K., Leung L.R., Zhang Y., Wang K., Fan J. (2018). Impacts of compound extreme weather events on ozone in the present and future. Atmos. Chem. Phys..

[59] Palmer P.I., Marvin M.R., Siddans R., Kerridge B.J., Moore D.P. (2022). Nocturnal survival of isoprene linked to formation of upper tropospheric organic aerosol. Science.

[60] Guenther A., Karl T., Harley P., Wiedinmyer C., Palmer P.I., Geron C. (2006). Estimates of global terrestrial isoprene emissions using MEGAN (Model of Emissions of Gases and Aerosols from Nature). Atmos. Chem. Phys..

[61] Ma M., Gao Y., Ding A., Su H., Liao H., Wang S., Wang X., Zhao B., Zhang S., Fu P. (2022). Development and Assessment of a High-Resolution Biogenic Emission Inventory from Urban Green Spaces in China. Environ. Sci. Technol..

[62] Friedl M.A., Sulla-Menashe D., Tan B., Schneider A., Ramankutty N., Sibley A., Huang X. (2010). MODIS Collection 5 global land cover: Algorithm refinements and characterization of new datasets. Remote. Sens. Environ..

[63] Liu Y., Li L., An J., Huang L., Yan R., Huang C., Wang H., Wang Q., Wang M., Zhang W. (2018). Estimation of biogenic VOC emissions and its impact on ozone formation over the Yangtze River Delta region, China. Atmos. Environ..

[64] Gao Y., Zhang J., Yan F., Leung L.R., Luo K., Zhang Y., Bell M.L. (2020). Nonlinear effect of compound extreme weather events on ozone formation over the United States. Weather. Clim. Extrem..

